# From pain to meningitis: bacteria hijack nociceptors to promote meningitis

**DOI:** 10.3389/fimmu.2024.1515177

**Published:** 2025-01-14

**Authors:** Huazhen Liu, Xingxing Kong, Yuqin Zeng, Jinyun Chen, Zhanpeng Chen, Lanlan Liu, Quan Ma, Xuhui Liu, Shuihua Lu

**Affiliations:** ^1^ National Clinical Research Center for Infectious Disease, Shenzhen Third People’s Hospital, Shenzhen, Guangdong, China; ^2^ Department of Tuberculosis, Shanghai Public Health Clinical Center, Fudan University, Shanghai, China; ^3^ Shanghai Public Health Clinical Center, Fudan University, Shanghai, China

**Keywords:** bacterial meningitis, nociceptor sensory neurons, TRPV1, CGRP, neuroimmune axis

## Abstract

Bacterial meningitis is a severe and life-threatening infection of the central nervous system (CNS), primarily caused by *Streptococcus pneumoniae* and *Neisseria meningitidis*. This condition carries a high risk of mortality and severe neurological sequelae, such as cognitive impairment and epilepsy. Pain, a central feature of meningitis, results from the activation of nociceptor sensory neurons by inflammatory mediators or bacterial toxins. These nociceptors, abundantly present in the meninges, trigger neuroimmune signaling pathways that influence the host immune response. However, the mechanisms by which bacteria hijack these nociceptors to promote CNS invasion and exacerbate the disease remain poorly understood. This review examines the interactions between bacteria and meningeal nociceptors, focusing on their direct and indirect activation via ion channels, such as transient receptor potential vanilloid-1 (TRPV1) and transient receptor potential ankyrin 1 (TRPA1), or through the release of neuropeptides like calcitonin gene-related peptide (CGRP). These interactions suppress immune defenses by inhibiting macrophage activity and neutrophil recruitment, thus facilitating bacterial survival and invasion of the CNS. Understanding this neuroimmune axis may open potential therapeutic targets for treating bacterial meningitis by enhancing host defenses and mitigating pain. Further research using advanced methodologies is essential to clarify the role of nociceptor-mediated immune modulation in this disease.

## 1 Introduction

Nociceptor sensory neurons serves as a detector of tissue damage and inflammatory response. After activation resulting from mechanical and chemical stimuli, and generate pain and release neuropeptides from their synaptic terminals, thereby regulating the immune response through the neuropeptides-immune axis (1). Nociceptor sensory neurons process pain signals differently from general sensations in the body, such as visual, auditory, and gustatory stimuli (2). Pain is a category of deep sensations that are found in the joints, bones and muscles to protects the body (3). The meninges are innervated by dense nociceptor sensory neurons. The meninges are highly relevant in bacterial meningitis, a life-threatening central nervous system (CNS) infection caused by bacteria such as Streptococcus pneumoniae and Neisseria meningitidis secondary to nasopharyngeal colonization, followed by mucosal invasion, bacteremia spread, and finally across the blood-brain barrier into the subarachnoid space (4). While bacterial meningitis is more common in children, the elderly, and immunosuppressed individuals, it can result in severe pain and other neurological symptoms (5). The condition carries a high risk of disability and death, with serious sequelae such as cognitive impairment, hearing loss, and epilepsy (6). Epidemiologically, bacterial meningitis remains a significant global health concern. The disease is prevalent worldwide but has a higher incidence in low- and middle-income countries (7). The “meningitis belt” in sub-Saharan Africa, stretching from Senegal to Ethiopia, experiences frequent outbreaks, primarily caused by Neisseria meningitidis. Other common pathogens include Streptococcus pneumoniae and Haemophilus influenzae type b (Hib). Factors such as overcrowding, seasonal climate changes, and socioeconomic conditions contribute to the spread of the disease (8). Despite the availability of vaccines, these regions continue to face challenges in controlling outbreaks.

Current prevention and treatment strategies for bacterial meningitis have notable shortcomings (9). Vaccination programs, while effective, suffer from uneven global coverage due to limited access, high costs, and logistical challenges in resource-poor settings. Additionally, the emergence of antibiotic-resistant bacterial strains complicates treatment efforts, making standard antibiotics less effective and necessitating the use of more potent, often less accessible medications. Early diagnosis is crucial but hindered by non-specific initial symptoms and the lack of rapid, affordable diagnostic tools, particularly in remote or under-resourced areas (10). These issues highlight the need for enhanced global vaccination efforts, improved diagnostic methods, and strengthened healthcare infrastructure to effectively reduce the burden of bacterial meningitis worldwide. Despite the critical need for research into bacterial meningitis, the mechanisms by which pain and neuroimmune interactions affect meningeal host defenses remain poorly understood. This review examines how bacteria activate nociceptors to signal afferent neurons and induce immune responses. Specifically, we discuss how bacteria use the brain neuron-immune signaling pathway to promote the survival of pathogens, as well as further understanding of neuroimmunomodulatory pathways to provide possible potential treatments for bacterial meningitis.

## 2 Bacteria activate meningeal nociceptors

Bacteria activate nociceptors, either directly or indirectly, through action potentials triggered on nociceptors, or pathogen-associated molecular pattern (PAMP) recognition. Gram-positive bacteria, such as Streptococcus pneumoniae can destroy cell membranes by producing hemolysin, leading to cell lysis and disruption of the blood-brain barrier. This damage allows bacterial invasion into brain tissue, causing meningitis. Hemolysin forms membrane pores by destroying cell membranes, leading to ion influx and the generation of action potentials, which activate nociceptors and result in abnormal pain during infection (11). In contrast, Gram-negative bacteria such as Neisseria meningitidis activate the transient receptor potential ankyrin 1 (TRPA1) ion channel on nociceptors by binding lipid A, a component of the lipopolysaccharide (LPS) membrane on the bacterial cell wall, to induce pain. The meningococcus successfully traversed the blood-brain barrier and gained access to the subarachnoid space (12, 13). Within this space, the arachnoid and pia meninges, which are comprised of a specific type of epithelial cell arrangement, serve as primary targets for meningococcal bacteria (14, 15). These pathogens employ transcellular pathways to breach a tightly interconnected layer of epithelial cells. Pial epithelial cells express various pattern recognition receptors (PRRs), including nociceptor toll-like receptor 4 (TLR4), nociceptor toll-like receptor 7 (TLR7), and nociceptor toll-like receptor 9 (TLR9), enabling them to recognize lipopolysaccharide (LPS) and CpG motifs present in Neisseria meningitidis DNA (16, 17). This recognition allows for the identification of pathogen-associated molecular patterns (PAMPs) within the CNS, triggering an immediate anti-pathogen response. Once identified, cytokines such as tumor necrosis factor (TNF), interleukin-1 (IL-1A), among others, significantly increase in concentration within the cerebrospinal fluid during bacterial meningitis cases. This indicates a crucial role played by IL-2 complex (CPLX) in initiating inflammatory responses. Meningococci exploit transcellular pathways to traverse a single layer of tightly connected epithelial cells. Meningococcal meningitis is characterized by elevated levels of pro-inflammatory cytokines like tumor necrosis factor α (TNF-α), interleukin-1β (IL-1β), and IL-6 within cerebrospinal fluid (CSF). Additionally, chemokines such as IL-8 (CXCL8) are recruited along with numerous white blood cells. Increased concentrations of growth-related oncogene α (CXCL1), monocyte chemoattractant protein 1 (MCP-1) (CCL2), macrophage inflammatory protein 1α (MIP-1α) (CCL3) and MI-1β (CCL4). CPLX and associated cells release diverse inflammatory cytokines and chemokines that attract neutrophil monocytes and T-cells towards infection sites while promoting phagocytosis for efficient bacterial clearance from these areas (13, 16). High concentrations of cytokines can be observed within cerebrospinal fluid samples obtain.

LPS also sensitizes transient receptor potential vanilloid-1 (TRPV1) through TLR4 nociceptors. Once activated, the TRPV1 nociceptor transmits signals to corticotropin-releasing hormone neurons to induce an acute stress response in the paraventricular hypothalamic nucleus. LPS amplifies neuroinflammatory responses by participating in nociceptor-specific receptors, such as G-protein-coupled receptor Formylpeptide receptor1 (FPR1), which detects bacterial flagellin, thereby enhancing pathogenesis and promoting bacterial transmission (18–21). Bacterial proteases or host cell proteases can regulate the excitability of dorsal root ganglion (DRG) neurons, increasing or decreasing their activity (22, 23). The Mycobacterium tuberculosis specific lipid glycoliptidyl thiopeptide-1 (SL-1) can target notional molecules to cause persistent cough in pulmonary tuberculosis (24, 25). Bacteria-induced immune mediators can also activate nociceptor sensory neurons, leading to pain and the release of neuropeptides. These mediators can further influence neural reflex circuits, including those involved in the immune response through the inflammatory reflex of the vagus nerve (26). The bacterial metabolite indole can also induce calcium responses to DRG neuron subpopulations, activate TRPA1 channels, and promote pain. Bacterial products can also affect afferent nerve signals in DRG, leading to spinal nerve pain (27). Immune cells T lymphocytes locally release enkephalins (endogenous opioids) that reduce pain caused by inflammation by down-regulating the activation of peripheral nociceptors, thereby maintaining intestinal homeostasis and normal behavior in mice (28). Nociceptors sense the innervation of barrier tissues that are constantly stimulated by microorganisms. During infection, pathogenic microorganisms can break through the barrier surface and produce pain by directly activating nociceptors (29). Activation of nociceptors and neurons in the brain may lead to neurogenic inflammation. Similar to macrophages, microglia produce potent pro-inflammatory cytokines that sensitize the meninges to nociceptors (30). The respiratory tract has intensive nociceptor innervation, and viral invasion triggers activation of sensory neurons during SARS-CoV-2 infection (31). Similarly, a bacteria-induced inflammatory response causes pain by directly activating injured neurons. After bacterial infection, ligand-gated ion channels and nociceptor receptors, such as TRPV1, are strongly upregulated, which can lead to hyperalgesia (32). TRPV1 sensory neurons modulate neutrophil activity in a tissue-specific manner. Therapeutic modalities targeting hurtful feelings may have a beneficial role in anti-infective therapy (33, 34).

### 2.1 Induce an immune response in the CGRP-RAMPL axis of the NA1.8 system

Pain-sensing primary afferent neurons densely innervating TRPV1 lead to increased leukocyte and neutrophil infiltration, accompanied by elevated levels of pro-inflammatory cytokines, promoting host immune overreaction (35). Allergen sensitization triggers IgE-producing plasma cells, further initiating and amplify allergic airway inflammation by through the Fcϵ R1-γ receptor expressed by nociceptors (36). TRPV1 (+) Nav1.8 (+) nociceptors regulate the IL-23/IL-17 pathway and control the skin immune response through interactions with dendritic cells (DDCs) (37). Increased local release of calcitonin gene-related peptide (CGRP) promotes additional release of CGRP from TRPV1. The presence of TRPV1 neurons inhibited the recruitment of neutrophils to the infected area, shifts macrophages toward an M2-like anti-inflammatory state, and inhibits macrophage polarization toward the pro-inflammatory M1 phenotype. This reduced the level of inflammation in the infected area, thus exacerbating local infection. TRPV1 neurons inhibit neutrophil recruitment and regulate macrophage polarization by releasing CGRP (38). These injurious afferent nerves promote an overactive host immune response (35). Nociceptor sensory neurons also inhibit neutrophil and gamma-delta T cell responses during bacterial lung infections and fatal pneumonia (39). Nociceptors promote macrophage aggregation by transmitting CGRP signals via the NA1.8 system and RAMP1 receptors, facilitating immune responses (4). Nociceptors are activated during bacterial infection, and when activated nociceptors release CGRP signals through immune cell receptors, inhibiting the ability of macrophages to clear bacteria. This neuroimmune axis suppresses host defense and exacerbates bacterial meningitis. Nociceptor neuronal ablation reduces invasion of the meninges and brain by two bacterial pathogens: Streptococcus pneumoniae and Streptococcus agalactis. Streptococcus pneumoniae activates nociceptors through its pore-forming toxin, porolysin, which triggers CGRP release from nerve endings. CGRP acts on receptor activity-modifying protein 1 (RAMP1) in meningeal macrophages, polarizing their transcriptional response, inhibiting macrophage chemokine expression, neutrophil recruitment, and dural antimicrobial defense. This process impairs the immune response and bacterial clearance in the meninges and brain. Bacteria hijack CGRP-RAMP1 signaling in meningeal macrophages to facilitate brain invasion. Targeting this neuroimmune axis in the meninges could boost host defenses and potentially lead to treatments for bacterial meningitis. Nociceptor sensory neurons activate the sensation of pain and trigger defensive behaviors to protect the organism. Bacterial infections induce pain through previously unknown molecular mechanisms, and bacteria can activate nocioreceptors nociceptors directly via immune responses mediated by TLR2, MyD88, T cells, B cells, neutrophils, and monocytes. Mechanical and thermal hyperalgesia in mice were associated with live bacterial load rather than tissue swelling or immune activation. Bacteria induce calcium fluxes and action potentials in nociceptor neurons through different mechanisms, including through bacterial N-formylated peptides and porotoxin-forming alpha-hemolysin. Ablating Nav1.8 spectrum neurons, including nociceptors, eliminates pain during bacterial infection, but simultaneously increases local immune infiltration and lymph node enlargement in the draining lymph nodes. These findings indicate that bacterial pathogens directly activate sensory neurons to regulate by directly activating sensory neurons that regulate inflammation, which is the nervous system in host-pathogen interactions (40). Activation of TRPV1 neurons induces a local Th17 immune response, boosting host defenses against Candida albicans and Staphylococcus aureus. The findings suggest that TRPV1 neuronal activation is sufficient to initiate host defense mechanisms and demonstrate the presence of functional anticipatory innate immunity at sites near infection, which relies on reverse neuronal activation (41). Calcitonin gene-related peptide (CGRP) is a 37-amino acid neuropeptide predominantly found primarily found in the pain nerve C and Aδ sensory fibers (42). CGRP is stored in dense-core vesicles at sensory nerve endings and is released via calcium-dependent exocytosis (33, 34). The release of CGRP occurs after activation of the TRPV1/TRPA1-CGRP-RAMP1 axis receptor. CGRP acts on neutrophils, monocytes, and macrophages through receptor activity modification protein 1 (RAMP1). To inhibit recruitment, accelerate death, enhance pinocytosis, and extremize macrophages into a pro-repair phenotype (43). Pain detected by nociceptors plays a critical role in injury response. TRPV1 activation triggers macrophage-dependent induction of osteopontin (Spp1) -expressing dermal fibroblasts. Epidermal abrasions induce SPP1-expressing dermal fibroblasts and hair growth via TRPV1 neurons and CGRP-dependent mechanisms. TRPV1 nociceptors coordinate the role of macrophage and fibroblast support (44). In TRPA1/TRPV1 nociceptor assays, nociceptor activation leads to damage of the mucosal lining, increased intestinal permeability, and altered transcriptional profiles of goblet cell markers, mucus regulation, immune response, and tight junction protein genes (45). Depletion of TRPV1 sensory nerves increases macrophage numbers and TNF-α production, the TRPV1 sensory nerve inhibits neutrophil and γδT cell recruitment through RAMP1 and SSTR5 signaling, and decreases the CCR2 macrophage response via RAMP1 signaling. Additionally, CCR2 macrophage response is increased through SSTR5 signaling (46). HRH1-mediated sensitization of TRPV1 has been linked to visceral hypersensitivity and abdominal pain in patients with IBS (47). Bacterial products can interact with nociceptor neurons during pathogen infection. Neurogenic inflammation is an integral component of pain signaling and has been recently implicated host-pathogen defense. Nociceptor neurons play a significant role in the inflammation induced by Bacillus anthracis and edema toxin, possibly affecting the pathogenesis of the bacteria (48).

Calcitonin gene-related peptide (CGRP) is a neuropeptide. This receptor complex consists of calciton receptor (CLR), receptor active modification protein 1 (RAMP1), and receptor component protein (RCP). RAMP1 is a molecular chaperone of the CLR, which binds to the CLR to form a complex that gives the CLR the ability to recognize and bind to CGRP. The primary mechanism of action of CGRP involves the activation of G protein-coupled receptors (GPCRS), which in turn activates adenylate cyclase, leading to elevated cAMP levels and activation of protein kinase A (PKA), a process that plays a key role in immunosuppression. Inhibition of leukocyte recruitment: CGRP can inhibit leukocyte recruitment, which is achieved by reducing the production and action of chemokines. Chemokines such as CCL2, CCL3 and TNF are key signaling molecules that guide white blood cells to move to inflammatory sites. CGRP reduces white blood cell recruitment by inhibiting the expression of these chemokines. Inhibition of macrophage response polarization to Streptococcus pneumoniae: In the case of Streptococcus pneumoniae infection, CGRP is able to inhibit the response of macrophages and reduce their phagocytosis and clearance of bacteria, which may be one of the mechanisms by which bacteria evade the host immune response (49). Inhibition of chemokines CCL2, CCL3 and TNF (50) by inhibiting the production of these chemokines, CGRP reduces the infiltration of white blood cells at the site of inflammation, thereby reducing the inflammatory response. Up-regulation of immunosuppressive transcription factors: CGRP is also able to up-regulate immunosuppressive transcription factors, such as cAMP response element regulator (CREM) and Jdp2, which further suppress the immune response. Streptococcus pneumoniae invades the central nervous system with toxins such as pneumonolysin (PLY) and cholesterol-dependent cytolysin. During this process, nocioreceptors signal immune cells in the meninges by releasing CGRP (49), a neuroimmune axis that suppresses host defenses and exacerbates bacterial meningitis. CGRP polarizes its transcriptional response via RAMP1 on meningeal macrophages, inhibiting macrophage chemokine expression, neutrophil recruitment, and dural antimicrobial defense. Pneumococcus, the most common and aggressive cause of bacterial meningitis, can induce a novel apoptosis-inducing factor dependent (AIF-dependent) form of brain cell apoptosis. Loss of production of two pneumococcal toxins, pneumolysin and H2O2 (49), eliminated mitochondrial damage and apoptosis. Purified pneumolysin or H2O2 induced apoptosis in microglia and neurons in vitro. Both toxins induce an increase in intracellular Ca2+ and trigger mitochondrial release of AIF. Chelating Ca2+ can effectively block AIF release and cell death (51, 52). Survivors of bacterial meningitis suffer from extensive neurological sequelae caused by neuronal cell damage. One trigger for this damage is the host inflammatory response. Pneumococcus directly induces damage to the human neuronal cell line, resulting in damage and activation of nociceptors that promote the release of CGRP in large numbers, Exacerbating the CGRP-RAMP1 axis-secreted factor nosioreceptor sensory neurons inhibiting neutrophil and gamma-delta T cell responses in bacterial lung infections and fatal pneumonia. Bacteria hijack CGRP-RAMP1 signaling in meningeal macrophages to promote brain invasion. In macrophages, the expression and expansion of biological processes associated with recruitment of white blood cells, including mediators that promote chemotaxis, such as Ccl12, Ccl2, Ccl3, and TNF, may coordinate the meninges’ protective brain toward CNS-associated phagocytes by recruiting immune cells that perform antimicrobial functions, such as neutrophils and monocytes. Including meningeal macrophages, meningeal MRC1 macrophages, no dendritic cells, monocytes, or neutrophils. Meningeal macrophages play a key role in host defense.

### 2.2 Nociceptors induce immune responses through the SP-NK1R axis

Nociception promotes the action of TRPV1 nociceptors through substance P (SP). SP specifically activates neutrophils via the neurokinin-1 receptor (NK1R) through the activation of neutrophils marker CD11b rise in integrin, as well as inducing neutrophil chemotactic factors such as alpha/CCL3 and MIP-1-2/CXCL2 at both the mRNA and protein levels. Additionally, the chemokine receptors CCR1 and CXCR2 are upregulated (53, 54). It has been documented that SP plays a protective role for the host-protective during sensory-injury-induced neuronal infections, independent of CGRP receptor signaling (55). The activation of neutrophils by SP is further evidenced by neutrophil activation markers. SP is involved in pial arteriole vasodilation during pneumococcal meningitis in rats, and can promote bacterial invasion. Neuropeptides, including SP, can dilate blood vessels (56). SP binding to NK1R promotes cell proliferation and migration (57). Pain sensing nerves or nociceptors sense local environmental changes and usually contain neuropeptides. Nociceptors amplify host responses such as calcitonin gene- related peptide (CGRP) or Substance P (SP) (58).

### 2.3 Induce immune response in VIP axis of NA1.8 system

Nociceptors are believed to contribute to inflammatory lung diseases, though the mechanisms involved remain unclear. In the lungs, nociceptors act as mediators of neuroimmune cross-talk. Nociceptors can reduce inflammation in asthmatic models. Vasoactive intestinal peptide (VIP) is expressed at high levels in pulmonary nociceptors, and when these nociceptors are stimulated with capsaicin in vitro, VIP is released from juvenile mouse nociceptors. VIP plays a critical role in mediating nociceptor-driven lung inflammation. During lung inflammation, nociceptors are activated and exacerbate the inflammatory response by releasing pro-inflammatory neuropeptides (59).

## 3 Meningeal nociceptive receptors stimulate the brain through synaptic transmission

The somatosensory nervous system is anatomically located within primary and secondary lymphoid tissues and mucous membranes, where it plays a direct role in regulating immunity. In response to danger signals, neurons release neuropeptides that initiate defense reflexes, chemotaxis, adhesion, and local infiltration of immune cells. The biology of nociceptor neurons provides valuable insights into identifying new immune drivers, addressing inflammation, and developing strategies to maintain homeostasis (60). Nociceptors in the extremities transmit signals from the first neuron in the spinal ganglion to the nucleus proprius in the posterior horn of the spinal cord, and from the second neuron to the ventroposterolateral nucleus of the thalamus in the third neuron. Neuropeptides influence neurotransmitter transmission by promoting their release through various mechanisms. Neuropeptides can promote the release of neurotransmitters in a variety of ways. This interaction affects nervous system function, including nerve signal transmission and regulation. The complex interactions between neurotransmitters and neuropeptides play a critical role in neuroregulation (47, 61).

## 4 Conclusions and future directions

**Figure 1:**
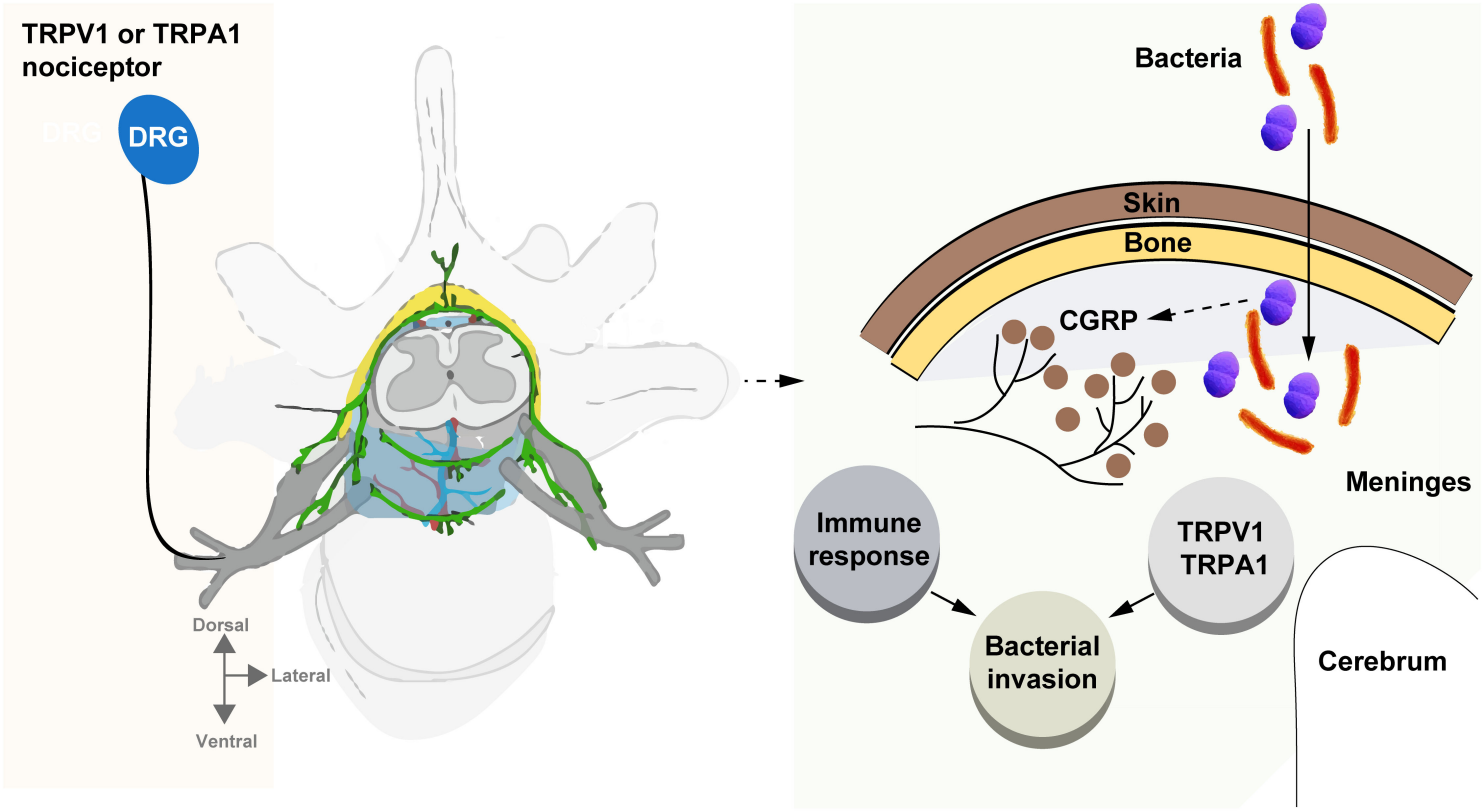
Bacteria hijack nociceptors to promote meningitis. This review underscores the crucial role of nociceptor neurons in bacterial meningitis, highlighting how pathogens exploit these neurons to invade the central nervous system (CNS) and drive disease progression. Through interactions with ion channels like TRPV1 and TRPA1, and the release of neuropeptides such as CGRP, nociceptors not only trigger pain but also modulate immune responses, aiding bacterial survival and CNS invasion. This neuroimmune interaction, which suppresses macrophage activity and neutrophil recruitment, presents a key challenge for treating bacterial meningitis.

This review emphasizes the critical role of nociceptor sensory neurons in the pathogenesis of bacterial meningitis, particularly in how bacterial pathogens exploit these neurons to promote central nervous system (CNS) invasion and disease progression. Nociceptors, through their interactions with ion channels such as TRPV1 and TRPA1 (**Figure 1**), and the release of neuropeptides such as calcitonin gene-related peptide (CGRP), not only contribute to the sensation of pain but also modulate immune responses in a manner that facilitates bacterial survival and CNS invasion. This neuroimmune crosstalk, particularly the suppression of macrophage activity and neutrophil recruitment, highlights the dual role of nociceptors in both pain generation and immune regulation, presenting a significant challenge for bacterial meningitis treatment.

The current understanding of nociceptor-mediated immune modulation suggests new therapeutic avenues, particularly by targeting the TRPV1-CGRP axis and other neuroimmune pathways. Inhibiting these pathways may enhance host defenses and reduce bacterial invasion, offering a dual benefit: mitigating both pain and infection. However, the complexity of this interaction, in which nociceptor activity simultaneously aids pathogen persistence while mediating pain, necessitates careful therapeutic balancing to avoid exacerbating either condition (**Figure 1**).

Several key areas of future research emerge from this review. First, further elucidation of the molecular mechanisms by which bacteria activate nociceptors and subsequently suppress immune functions is crucial. In particular, more detailed investigations into the signaling pathways of TRPV1 and TRPA1 ion channels and their roles in immune suppression may provide insights into targeted interventions. Additionally, identifying other neuroimmune interactions, beyond the TRPV1-CGRP axis, is necessary to fully understand the breadth of bacteria-neuron-immune cross-talk during CNS infections (**Figure 1**).

Second, future studies should focus on developing animal models that more accurately replicate human bacterial meningitis, particularly in the context of nociceptor involvement. Advanced techniques, such as single-cell RNA sequencing and in vivo imaging could provide deeper insights into how bacterial products modulate nociceptor function during infection.

Lastly, exploring the potential of combinatorial therapies that target both nociceptive pathways and immune responses holds promise. Combining neuroimmune modulation with antimicrobial therapies could enhance bacterial clearance while alleviating pain, thereby improving outcomes for patients with bacterial meningitis. Moreover, integrating immunotherapy approaches that restore the immune system’s ability to respond to bacterial invasion without triggering excessive neurogenic inflammation may further refine treatment strategies.

Given the dual role of nociceptors in both pain generation and immune suppression, drugs that target these pathways, particularly those modulating TRPV1 and CGRP signaling, could offer a therapeutic approach. By blocking or modifying these pathways, it may be possible to reduce pain and enhance the immune response against bacterial invasion in the CNS. Current research has also pointed to the possibility of using these neuroimmune interactions as a target for novel treatments. For example, therapies that inhibit the CGRP-RAMP1 axis might prevent bacteria from evading immune defenses, thus improving bacterial clearance. However, these interventions must be carefully balanced, as inhibiting nociceptor activity might also impact other essential pain responses. In conclusion, while there is promising potential for drugs targeting nociceptors to be integrated into bacterial meningitis treatment strategies, further research is required to clarify the molecular pathways involved and ensure that such interventions do not inadvertently impair the host’s immune response.

In summary, understanding the complex interactions between nociceptor neurons and the immune system during bacterial meningitis opens new frontiers for therapeutic innovation. As our knowledge of this neuroimmune axis grows, so too does the potential for more effective treatments for this devastating disease.
